# MICA-129 Met/Met contributes to the susceptibility of colorectal cancer

**DOI:** 10.1097/MD.0000000000046637

**Published:** 2025-12-19

**Authors:** Jingxin Ye, Renan Chang, Jianfeng Zhang, Fei Qian, Weifeng Ding

**Affiliations:** a Department of Gastroenterology, The Affiliated Suqian Hospital of Xuzhou Medical University, Suqian, Jiangsu Province, China; b Department of Laboratory Medicine, Affiliated Hospital of Nantong University, Medical School of Nantong University, Nantong, Jiangsu Province, China; c Department of Hepatic-Biliary-Pancreatic Surgery, Affiliated Hospital of Nantong University, Medical School of Nantong University, Nantong, Jiangsu Province, China; d Department of Gastroenterology, Affiliated Hospital of Nantong University, Nantong, Jiangsu Province, China; e Department of Gastrointestinal Surgery, Affiliated Hospital of Nantong University, Medical School of Nantong University, Nantong, Jiangsu Province, China.

**Keywords:** cancer immunity, colorectal cancer, immunosurveillance, MICA-129 dimorphism, NKG2D

## Abstract

The highly polymorphic human major histocompatibility complex class I chain-related gene A (MICA) regulates immune surveillance and destroys tumor cells by activating its receptor, the natural killer group 2D. This study aimed to examine a single nucleotide polymorphism of this gene at codon 129 (MICA-129) in association with colorectal cancer (CRC). Using PCR sequencing, the MICA-129 polymorphism was examined in 104 patients with CRC and 536 healthy controls. Specific MICA-129 single nucleotide polymorphism was analyzed for its association with CRC susceptibility, clinical phenotypes, and selected CRC-associated microsatellite instability, driver gene mutation, immune checkpoint programmed death ligand 1, and diagnostic biomarkers carbohydrate antigen 19-9 and carcinoembryonic antigen. The MICA-129 heterozygous A/G (Met/Val) genotype was associated with less aggressive clinical characteristics, such as a reduced prevalence of the ulcerated subtype (*P* = .0489, OR = 0.59) and lymph node involvement (*P* = .0217, OR = 0.46). Conversely, the MICA-129 homozygous allele A/A (Met/Met) variant was related to more advanced clinical characteristics, such as increased tumor invasion depth (T3/4; *P* = .0261, OR = 2.10), driver gene mutation (*P* = .0363, OR = 2.65), and KRAS mutation (*P* = .0392, OR = 2.23). Additionally, patients with carbohydrate antigen 19-9-positive CRC had a lower MICA-129 Met/Val variation, whereas those with carcinoembryonic antigen-positive CRC had a higher MICA-129 Met/Met variant (*P* = .0330/OR = 0.22 and *P* = .0034/OR = 2.99, respectively).

## 1. Introduction

Colorectal cancer (CRC), a malignant tumor, has the third highest incidence and mortality in African Americans^[1]^ and globally.^[2]^ In tumors such as CRC, tumor immunotherapy, particularly checkpoint blockade therapy, has demonstrated remarkable results.^[3–6]^ The primary objectives of tumor immunotherapy are to restore the normal function of immune cells that infiltrate tumors and to stimulate the immune surveillance function in patients with cancer.

The natural killer group 2A (NKG2A), one of the 5 members of the NKG2/CD94 family, is a novel checkpoint inhibitory gene that enhances T and NK cell function to promote antitumor immunity.^[5,6]^ Approximately 21% sequence homology between NKG2A and the natural killer group 2D (NKG2D) exist, the latter being an important member of the NKG2/CD94 family.^[7]^ By activating its ligands, it effectively rejects tumor cells, particularly in the early stages of tumor growth.^[8,9]^ The highly polymorphic gene MICA encodes the major histocompatibility complex class I chain-related gene A (MICA), a high-affinity ligand of NKG2D.^[10]^ It exists in both membrane-bound and soluble isoforms.^[11]^ Persistent surface expression of MICA and shedding of soluble major histocompatibility complex class I chain-related gene A (sMICA)^[12,13]^ by late-stage human tumors might have a detrimental effect on local and systemic immune responses, which in turn promotes tumor immune evasion.^[14]^ However, it remains unknown what processes underlie the defective MICA biology of cancers.

Owing to its potential to impact MICA biology, the MICA-129 dimorphism has raised substantial research interest.^[15–19]^ The MICA alleles are classified as either strong (MICA-129Met) or weak (MICA-129Val) binders to the NKG2D receptor because of a substitution of methionine (Met) for valine (Val) at codon 129 of the α2 heavy chain domain.^[20]^ The MICA-129 Val/Val variant is associated with significantly higher sMICA levels^[21]^ and the progression of multiple myeloma. sMICA, combined with the patient’s MICA genotype, can serve as a definitive prognostic indicator of multiple myeloma.^[22]^ Similarly, the MICA-129 Val allele influences individual susceptibility for breast cancer development in Tunisian women.^[23]^ However, the association between MICA-129 polymorphism and CRC in the Chinese population remains unexplored. Therefore, we investigated whether MICA-129 polymorphisms, such as microsatellite instability (MSI), the clinical phenotype, and driver gene mutations, may impact MICA release and/or function during CRC progression.

## 2. Materials and methods

### 2.1. CRC patients and controls

This case–control study involved 104 patients with CRC and 536 healthy controls. Between January 2020 and December 2022, 104 patients with CRC were consecutively recruited from the Department of General Surgery at the Affiliated Hospital of Nantong University, Jiangsu Province, China.^[24]^ The inclusion criteria were as follows: newly diagnosed and histopathologically confirmed primary colorectal adenocarcinoma; classified according to the tumor node metastasis/UICC (Union for International Cancer Control) staging system; self-reported Southern Han Chinese ancestry; and no history of chemotherapy or radiotherapy before sample collection.^[25]^ The exclusion criteria were as follows: patients with a history of other malignant tumors; those with familial adenomatous polyposis or hereditary nonpolyposis CRC; those with concurrent inflammatory bowel disease.

The control group comprised 536 healthy individuals recruited from a Chinese population genetics study project at Fudan University, Shanghai, China. To ensure genetic background matching with the patient group, only controls who met the following criteria were included: healthy individuals without clinical evidence or history of any cancer during recruitment; recruited from hospitals and clinics across southern cities of China; and self-reported Southern Han Chinese ancestry. The exclusion criterion was a history of any type of cancer. The control group included 273 men (51%) and 263 women (49%), with a mean age of 46 years. To account for any confounding effects, the mean age and sex distribution between cases and controls were recorded and adjusted for in the subsequent analyses. All procedures in this study were approved by the Institutional Review Board of Affiliated Hospital of Nantong University (No. 2018-K008), and informed consent was obtained from all patients.

### 2.2. MICA dimorphism detection by PCR sequencing

Genomic DNA was extracted from peripheral blood cells of the controls and colorectal tumor tissues of the patients, respectively. PCR sequencing of the MICA gene’s exons 2 and 3 suggested the MICA-129 polymorphism.

All sequencing processes were conducted using a robotic automation system for DNA typing quality control to minimize sample mislabeling and misplacement. Each sample plate consisted of blind duplicates.

### 2.3. Screening for tumor microsatellite instability and KRAS, NRAS, BRAF oncogene mutations

Fluorescence in situ hybridization was used to analyze tumor MSI and detect MLH1, MSH2, MSH6, and PMS2.^[26]^ Microsatellite stability (MSS or CIN) was defined as the positive expression of all 4 genes; MSI was defined as the positive expression of 3 or fewer genes.

Tumor DNA was detected for mutations in codons 12, 13, 59, 61, 117, and 146 of the KRAS gene, NRAS, and the mutation in codon 600 of the BRAF gene^[26]^ by Fast Target next-generation sequencing.^[27]^

### 2.4. Statistical analysis

Gene counting under the assumption of homozygosity was used to establish haplotype gene frequencies. Correlations to histopathological parameters were conducted by comparing MICA alleles with tumor invasion, lymph node involvement, histological type, distant metastasis, UICC stages, microsatellite instability, driver gene mutation, immune checkpoint programmed death ligand 1 (PD-L1), and diagnostic biomarkers. From 2 × 2 tables of allele counts, exact *P* values (Fisher test or Mantel–Haenszel stratification test) were generated. Statistical significance was defined as a *P*-value <.05. The SPSS statistical software (version 20.0; IBM Corp., Armonk) was used for statistical analysis, including logistic regression. The association analysis was conducted using multivariate logistic regression models, with adjustment for age and sex, to account for any potential confounding effects caused by significant differences in age and sex between the case and control groups.

## 3. Results

### 3.1. The MICA-129 dimorphism differed significantly between patients and controls

Compared with 15 patients with CRC (14.42%) who exhibited the MICA-129 A/A allele (Met/Met), 54 (51.92%) exhibited the MICA-129 G/G allele (Val/Val) (*P* = .0000), and 35 (33.65%) exhibited the MICA-129 dimorphism A/G (Met/Val) (*P* = .0012). Compared with 48 controls (8.96%) who exhibited the MICA-129 A/A variant, 259 (48.32%) exhibited the MICA-129 G/G genotype (*P* = .0000), and 229 (42.73%) exhibited the MICA-129 A/G genotype (42.73%) (*P* = .0000).

Table 1 shows the clinical phenotype data for 104 patients with CRC. According to the 2X2 table analysis, the MICA-129 dimorphism A/G (Met/Val) was significantly associated with the ulcerated type, lymph node involvement, and late UICC stage of CRC, the control group (*P* = .0489, .0217, and .0169, respectively) (Table 2). Therefore, the Met/Val genotype may be associated with a more aggressive tumor phenotype rather than being a protective factor.

**Table 1:** The clinical information of CRC patients.

Clinical characteristics	Number	Clinical characteristics	Number
Age (yr old)		Invasion depth	
<60	23	T 1/2	24
≥60	81	T 3/4	76
Gender		Lymph node involvement	
Male	71	N0	55
Female	33	N1-3	47
Tumor size (cm)		Distance metastasis	
≤3	35	M0	93
>3	69	M1	11
Differentiation degree		UICC stage	
High	8	I/II	53
Medium	81	III/IV	51
Low	14		

CRC = colorectal cancer, UICC = Union for International Cancer Control.

**Table 2:** Comparison of the MICA-129 dimorphism to the phenotype of CRC patients.

	129 A	129 G SQ	129 A/G (%)	*χ* ^2^	*P* value	OR (95% CI)
**Gross classification**						
Protruded (n = 30)	5	12	13			
Ulcerated (n = 72)	10	40	22 (30.56)	3.88	.0489	0.59
Control (n = 536)	48	259	229 (42.72)			
**Lymph node involvement**						
N0 (n = 55)	7	25	23			
N1–3 (n = 47)	8	27	12 (25.53)	5.27	.0217	0.46
Control (n = 536)	48	259	229 (42.72)			
**UICC stage**						
I/II (n = 53)	7	24	22			
III/IV (n = 51)	8	30	13 (25.49)	5.71	.0169	0.46
Control (n = 536)	48	259	229 (42.72)			
**Serum biomarker**						
CA19-9 Neg (n = 54)	11	40	31 (37.8)	2.93	.0872	0.27
CA19-9 Pos (n = 44)	3	9	2 (14.3)	4.53	.0330	0.22
Control (n = 536)	48	259	229 (42.72)			

CA19-9 = carbohydrate antigen 19-9, CRC = colorectal cancer, UICC = Union for International Cancer Control.

To further evaluate the association between the MICA-129 polymorphism and CRC diagnostic biomarkers, the dependent linkage between the MICA-129 polymorphism and carbohydrate antigen 19-9 (CA19-9), carcinoembryonic antigen (CEA), and CYFRA21-1 (serum biomarkers) was investigated. Table 2 suggests that CA19-9 positive patients with CRC had a significantly higher genotype of MICA-129 dimorphism A/G than healthy controls (*P* = .033). In contrast with CA19-9 negative patients with CRC, the MICA-129 dimorphism did not reach statistical significance (*P* = .087), but it demonstrated a tendency towards a connection with CA19-9 positive status in patients with CRC.

### 3.2. The MICA-129 A/A (Met/Met) is a risk factor for CRC

The MICA-129 A/A allele (Met/Met) was associated with the tumor invasion depth of T3/4 in patients with CRC than healthy controls (*P* = .0261, Table 3). CEA-positive patients with CRC had a higher MICA-129 A/A allele (Met/Met) than healthy controls (*P* = .0034, Table 3). Similarly, the MICA-129 A/A allele (Met/Met) was higher in CEA-positive patients with CRC than in CEA-negative ones (*P* = .0648, Table 3).

**Table 3:** The MICA-129 A/A (Met/Met) associated with CEA positive and tumor invasion depth of CRC.

MICA-129	129 A (%)	129 G	129 A/G	*χ* ^2^	*P* value	OR (95% CI)
**Tumor invasion**						
T1/2 (n = 24)	2	11	11			
T3/4 (n = 76)	13 (17.11)	39	24	4.93	.0261	2.10
Control (n = 536)	48 (8.96)	259	229			
**Serum biomarker**						
CEA neg (n = 54)	5 (9.21)	28	21	3.39	.0648	2.88
CEA pos (n = 44)	10 (22.7)	22	12	8.57	.0034	2.99
Control (n = 536)	48 (8.96)	259	229			

CEA = carcinoembryonic antigen, CRC = colorectal cancer.

### 3.3. The MICA-129 A/A (Met/Met) is associated with the KRAS mutation subtype of CRC

CRC tumorigenesis involves the mutation of driver genes, such as KRAS and BRAF. Both patients with driver gene mutations and patients with KRAS codon 12 mutations had higher MICA-129 A/A allele (Met/Met) than healthy controls (*P* = .0363 and 0.0392, respectively) (Table 4). However, MICA-129 alleles were not associated with MSI, PD-L1, and CYFRA21-1 (Table 5).

**Table 4:** The MICA-129 A/A (Met/Met) associated with KRAS mutation subtype of CRC.

MICA-129	129 A (%)	129 G	129 A/G	*χ* ^2^	*P* value	OR (95% CI)
Driver gene mutation (n = 50)	9 (18.00)	22	19	4.383	.0363	2.65
KRAS codon 12 mutation (n = 29)	6 (20.69)	11	12	4.261	.0392	2.23
Control (n = 536)	48 (8.96)	259	229			

CRC = colorectal cancer.

**Table 5:** The MICA-129 SNP was not associated with MSI, PD-L1, and CYFRA21-1.

MICA-129	129 A (%)	129 G (%)	129 A/G (%)
PD-L1 pos (n = 9)	0 (0.00)	6 (66.67)	3 (33.33)
MSI (n = 13)	3 (23.08)	5 (38.46)	5 (38.46)
CYFRA21-1 pos (n = 37)	6 (16.22)	15 (40.54)	16 (43.24)
Control (n = 536)	48 (8.96)	259 (48.32)	229 (42.72)

MSI = microsatellite instability, PD-L1 = programmed death ligand 1.

**Figure 1.**
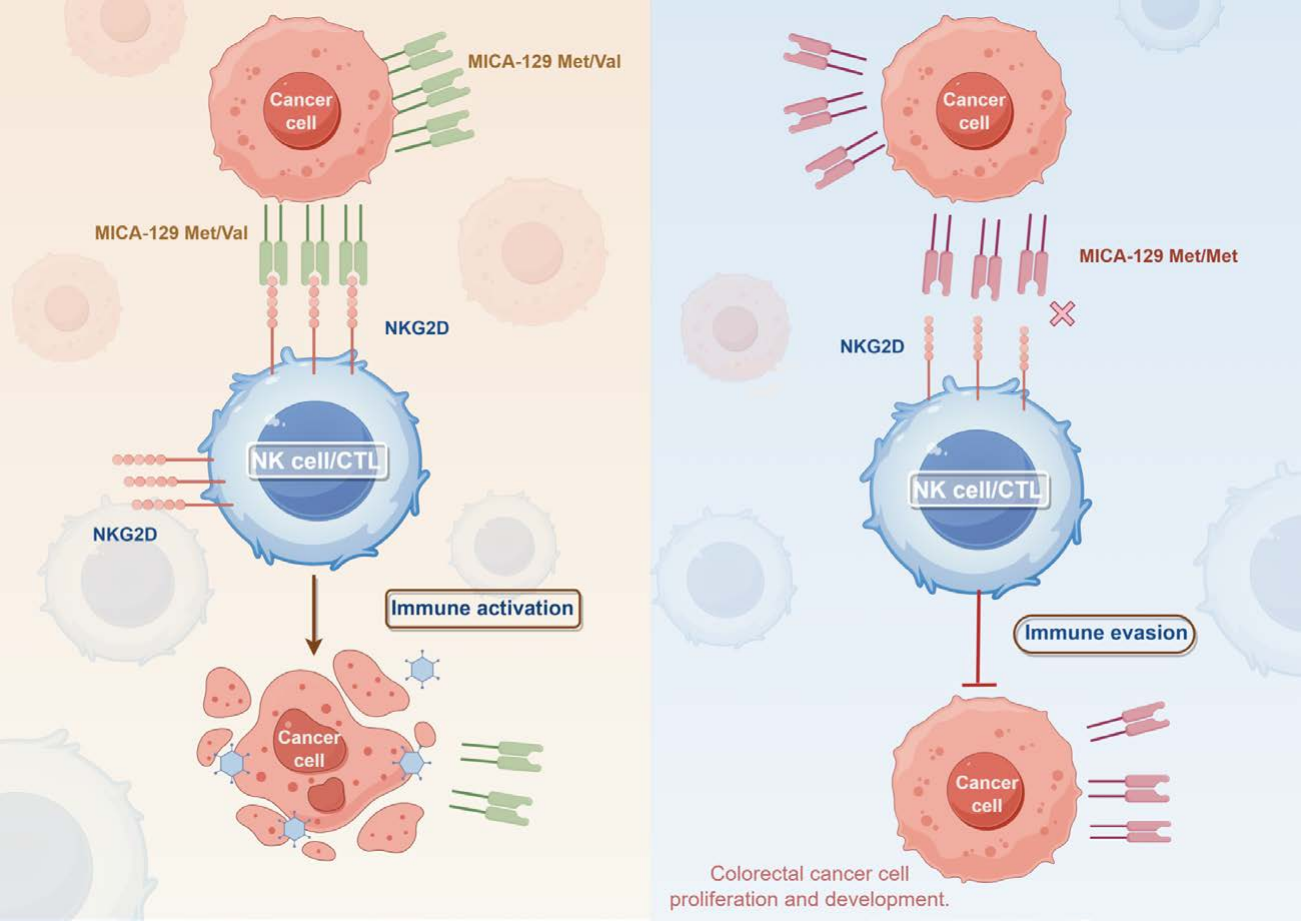
Proposed mechanism of action for the MICA-129 gene polymorphism in modulating CRC progression and immune surveillance. Solid line with arrow indicated induction, solid line with flat head indicated inhibition (created by Figdraw). CRC = colorectal cancer.

NKG2D-mediated immune activation is compromised in patients with the Met/Met genotype. Tumor cells can multiply and spread because of immune evasion caused by this disruption in immune signaling. This mechanism explains the associations between the Met/Met genotype and advanced disease characteristics, such as deeper tumor invasion (T3/4), lymph node involvement, late UICC stage, and driver gene mutations (Fig. 1).

## 4. Discussion

MICA serves as a key immune regulatory gene for immune surveillance by binding to its receptor NKG2D, which is commonly expressed as a tumor-associated antigen on the surface of tumor cells.^[28]^ However, through MICA shedding, polymorphism, or other mechanisms, transformed malignant cells can evade immunosurveillance and NK or CD8 T cell death.^[29]^ The MICA-129 polymorphism can influence the progression of various tumors, such as multiple myeloma,^[22]^ breast cancer,^[15]^ and nasopharyngeal cancer.^[30]^ In this study, lymph node involvement and late stage of CRC were significantly associated with patients carrying the MICA-129 Val/Val or Met/Met allele, but not the MICA-129 Met/Val allele. Tumor invasion depth, KRAS or driver gene mutations were associated with patients carrying the MICA-129 Met/Met genotype.

Additionally, the relationship between MICA-129 polymorphism and the protein expression level of PD-L1 (tumor immunotherapy inhibitor) and CRC’s MSI was investigated; however, no association was observed. Therefore, MICA-129 polymorphism is not an alternative to PD-L1 checkpoint inhibition in terms of tumor mutation burden.^[31]^

CA19-9 and CEA are 2 frequently used biomarkers for the therapeutic prognosis of CRC. Patients with CA19-9 positive patients had lower MICA-129 heterozygous A/G allele (Met/Val), whereas CA19-9 positive patients had higher MICA-129 homozygous A/A allele (Met/Met). Thus, the homozygous A/A of MICA-129 had a poor prognosis for CRC, whereas the heterozygous genotype was expected to have a favorable prognosis.

Taken together, the MICA-129 Met/Val variant was lower in the patients with CRC who had ulcerated subtype, lymph node involvement, and late stage (III/IV). Patients with high tumor invasion depth (T3/4), driver gene mutation, and KRAS mutation had a higher MICA-129 Met/Met genotype. To place these findings in a global context, they were compared with a study conducted in a genetically distinct Tunisian population. This comparison was conducted to explore the consistency of the MICA-129 polymorphism’s role in CRC across different ethnic and genetic backgrounds. Notably, the MICA-129 Val/Val genotype was identified as a risk genotype and a poor prognostic biomarker in the Tunisian population.^[15]^ This discrepancy suggests that the association between MICA-129 and CRC susceptibility varies across populations and is affected by differing haplotype structures, regional environmental factors, or interactions with other unidentified genetic variants. To differentiate between population-specific and universally relevant mechanisms, this research necessitates confirming genetic risk factors in varied ethnic groups.

However, this study has some limitations. First, the relatively small sample size, particularly in the patient subgroup analyses, may have restricted the statistical power to detect weaker associations; furthermore, it increased the risk of type II errors. Second, age and sex distribution differed between the CRC and control groups. Despite statistically adjusting for these variables in our analyses, residual confounding may still exist. Third, this study lacks longitudinal follow-up and survival data. Consequently, the prognostic value of the MICA-129 polymorphism for patient outcomes, such as overall or recurrence-free survival, could not be evaluated. Finally, all participants were recruited from a single geographic region and had Southern Han Chinese ancestry, which limits the generalizability of these findings to other ethnic or geographic populations. To confirm and broaden these findings, larger, prospective, multicenter designs and more diverse cohorts are warranted. Despite these limitations, this study provides novel insights into how the MICA-129 polymorphism contributes to CRC in the Southern Han Chinese population. The well-characterized patient cohort, histopathologically confirmed diagnoses, and analyses of associations with specific clinical phenotypes strengthen the internal validity of these findings.

## 5. Conclusions

The MICA-129 polymorphism is significantly associated with clinicopathological characteristics of CRC. The Met/Val genotype is associated with less aggressive disease presentation, whereas the Met/Met genotype is associated with markers of advanced and aggressive tumors. Collectively, MICA-129 may serve as a biomarker for tumor behavior in CRC.

## Acknowledgments

The work was supported by the Natural Science Foundation Project of Suqian Science and Technology and Technology Bureau (Grant No. K202412), Postdoctoral Science Foundation of China (Grant No. 2019M651930), and Jiangsu Province’s Key Young Medicine Talents Program (Grant No. QNRC2016688). We thank Bullet Edits Limited for the linguistic editing and proofreading of the manuscript.

## Author contributions

**Data curation:** Jianfeng Zhang, Fei Qian.

**Funding acquisition:** Jingxin Ye, Weifeng Ding.

**Investigation:** Jingxin Ye, Renan Chang, Jianfeng Zhang.

**Resources:** Jingxin Ye, Weifeng Ding, Renan Chang.

**Software:** Weifeng Ding, Fei Qian.

**Writing – review & editing:** Jingxin Ye, Weifeng Ding.
